# Lymphangiogenesis-related gene signature–based risk model for prognostic assessment of cervical cancer: immune–metabolic characterization and molecular subtype analysis

**DOI:** 10.3389/fgene.2026.1835653

**Published:** 2026-08-31

**Authors:** Xijing Fan, Lu Chen, Jing Xu, Shunjie Zheng, Qi Pan, Yifei Hu

**Affiliations:** 1 Department of Clinical Laboratory, Affiliated Jinhua Hospital, Zhejiang University School of Medicine, Jinhua, Zhejiang, China; 2 Department of Healthcare, Jinhua Maternal and Child Healthcare Hospital, Jinhua, Zhejiang, China; 3 Department of Gynecology, Jinhua Maternal and Child Healthcare Hospital, Jinhua, Zhejiang, China; 4 Lighthouse Research Institute, University of Nottingham Ningbo China, Ningbo, Zhejiang, China

**Keywords:** cancer, cervical cancer, lymphangiogenesis, molecular subtype, prognostic signature

## Abstract

**Background:**

Lymphangiogenesis promotes tumor dissemination and may shape the immune contexture of cervical cancer, yet lymphangiogenesis-related prognostic stratification and its immunometabolic implications remain insufficiently defined in cervical squamous cell carcinoma and endocervical adenocarcinoma (CESC).

**Methods:**

TCGA-CESC transcriptomes and clinical data were obtained from UCSC Xena and integrated with normal cervix tissues from the Genotype-Tissue Expression Project after batch correction. Prognostic LYMRGs were first identified from the differentially expressed set using univariable Cox proportional hazards analysis. Candidate genes were then reduced using an L1-regularized Cox model (Least Absolute Shrinkage and Selection Operator), and the remaining markers were entered into a multivariable Cox regression to obtain the final coefficients and compute an individualized risk score. The model’s prognostic value was further assessed in an independent Gene Expression Omnibus dataset. In addition, expression patterns of the signature genes were leveraged for molecular subtyping of TCGA samples via non-negative matrix factorization (NMF). Immune infiltration and immunotherapy-associated characteristics were interrogated through a multi-algorithm strategy (single-sample gene set enrichment analysis, CIBERSORT, ESTIMATE, Tumor Immune Dysfunction and Exclusion (TIDE), and Immunophenoscore . Additional analyses included pathway enrichment (GSEA/GO/KEGG), drug sensitivity prediction (pRRophetic/CellMiner), and ceRNA network analysis.

**Results:**

A six-gene LYMRG signature robustly stratified survival. High-risk patients had significantly worse overall survival in The Cancer Genome Atlas with AUCs of 0.819/0.801/0.801 at 1/3/5 years, and in GSE52903 (P = 0.001) with AUCs of 0.733/0.719/0.725. NMF identified two subtypes with distinct prognosis (P = 0.01) and divergent immune landscapes. Risk groups and subtypes exhibited consistent differences in immune infiltration, checkpoint expression, TIDE/IPS patterns, and pathway enrichment. Predicted chemosensitivity differed by risk group, and the ceRNA network suggested candidate upstream lncRNA regulators of the signature.

**Conclusion:**

A lymphangiogenesis-related six-gene model enables clinically meaningful prognostic stratification of CESC and links lymphangiogenesis programs to distinct tumor immune phenotypes and therapeutic vulnerabilities.

## Highlights

- A six-gene lymphangiogenesis-related signature (EFNA1, FASN, NRP1, ZIC2, IL1B, FOXP3) robustly stratified CESC prognosis, achieving 1/3/5-year AUCs of 0.819/0.801/0.801 in TCGA and 0.733/0.719/0.725 in GSE52903.
- NMF identified two molecular subtypes with distinct survival (P = 0.01) and divergent immune landscapes, supported by consistent differences in CIBERSORT/ssGSEA/ESTIMATE scores, checkpoint expression, and TIDE/IPS immunotherapy-related metrics.
- Risk-associated pathways and therapeutic vulnerabilities were characterized: high-risk tumors were enriched for ECM–receptor interaction/focal adhesion programs, showed risk-stratified predicted drug sensitivity, and a ceRNA network suggested candidate upstream lncRNA regulators of the signature.

## 1 Introduction

Cervical cancer continues to impose a substantial global health burden, contributing markedly to morbidity and mortality among women. In 2020, an estimated 604,000 new cases and 342,000 deaths were recorded worldwide (Wang et al., 2023; Singh et al., 2023). Cervical squamous cell carcinoma and endocervical adenocarcinoma (CESC) are the predominant histologic subtypes, with squamous cell carcinoma accounting for approximately 80% of cases (Cibula et al., 2023). Despite advances in prevention (HPV vaccination) and early detection, treatment options for advanced or recurrent disease are limited and outcomes remain poor (Wang J. et al., 2025; Zhang et al., 2025). Tumor metastasis and recurrence are the primary drivers of cervical cancer mortality (Liang et al., 2022), underscoring the need for better prognostic tools to identify high-risk patients.

Cervical cancer spreads early via the lymphatic route—pelvic lymph node metastasis (LNM) is a hallmark of aggressive disease and is closely linked to prognosis (Xia et al., 2025). Patients without nodal metastases have 5-year survival rates around 90%, whereas survival drops to approximately 35% with extensive nodal involvement (Tax et al., 2015). This clinical impact is reflected in the 2018 FIGO staging, which now classifies any nodal metastasis as stage IIIC, highlighting the prognostic weight of LNM (Bhatla et al., 2019). Tumor lymphangiogenesis—the formation of new lymphatic vessels—is pivotal for nodal spread. Tumor cells and stromal cells in the tumor microenvironment (TME) secrete pro-lymphangiogenic factors (e.g., VEGF-C, VEGF-D, angiopoietins) that stimulate lymphatic endothelial growth and vessel sprouting (Dadafarin et al., 2021; Solis-Castillo et al., 2020). Increased lymphatic vessel density and lymphatic invasion in primary tumors have been associated with higher risk of nodal metastases and worse outcomes (Tantari et al., 2022; Huang et al., 2023). For instance, fatty acid synthase (FASN) has been recently shown recently shown to promote cervical cancer cell migration and lymphangiogenesis, thereby driving LNM and poor survival (Du et al., 2022). Similarly, emerging evidence indicates that oncogenic factors like DDX24 and PRDX3 have been reported to orchestrate lymphangiogenesis (via secreted mediators such as AGRN or NF-κB signaling) to facilitate lymphatic spread (Wang B. et al., 2025; Wen et al., 2025). However, other studies in early-stage disease have yielded nuanced findings—Morotti et al. found that classical lymphangiogenesis markers (D2-40, VEGF-C) did not strongly correlate with nodal metastasis in early tumors, suggesting that pre-existing peritumoral lymphatics (rather than extensive new growth) might suffice for tumor spread (Tantari et al., 2022). These observations underscore a gap in understanding: while lymphatic dissemination is clearly critical, the specific molecular drivers linking lymphangiogenic activity to prognosis in CESC remain to be fully elucidated.

In this context, we aimed to develop and validate a novel prognostic signature in CESC based on lymphangiogenesis-related genes (LYMRGs). Distinct from previous studies that focused primarily on survival prediction, our study integrates a lymphangiogenesis-related six-gene signature with non-negative matrix factorization (NMF)-based molecular subtyping and immune–metabolic characterization, providing a multi-dimensional systems-biology framework to understand CESC progression. We hypothesize that this signature not only stratifies patient survival risk more accurately than existing models, but also correlates with the tumor immune microenvironment and molecular subtypes, thereby offering insights into underlying biology. Here we present the lymphangiogenesis-related six-gene prognostic signature for CESC and discuss its performance, biological significance, and potential clinical utility. We also explore the mechanisms linking these signature genes to tumor lymphatics and immunity—including the intriguing role of the m^6A^ RNA demethylase ALKBH5—and how our findings might inform future therapeutic strategies.

## 2 Methods

### 2.1 Data collection

RNA-seq expression matrices and matched clinical annotations for the TCGA cervical squamous cell carcinoma and endocervical adenocarcinoma cohort (TCGA-CESC; tumor n = 304, normal n = 3) were retrieved from UCSC Xena (https://xena.ucsc.edu/). Normal cervix transcriptomes were collected from the Genotype-Tissue Expression (GTEx) project (n = 10). To enable integrated analyses, The Cancer Genome Atlas (TCGA) and GTEx expression matrices were merged and batch effects were removed using the sva package (ComBat). Patients with an overall survival time <30 days, as well as those lacking survival information, were excluded, leaving 273 TCGA-CESC tumor samples for subsequent modeling. An independent cohort (GSE52903) was downloaded from the Gene Expression Omnibus (https://www.ncbi.nlm.nih.gov/geo/) and used as an external validation dataset (n = 51). Notably, GSE52903 is a gene expression microarray dataset (GPL6244). Lymphangiogenesis-related genes were collected from GeneCards (https://www.genecards.org/) and relevant published studies, resulting in a total of 660 lymphangiogenesis-related genes (Supplementary Table S1).

### 2.2 Identification of prognostic differentially expressed LYMRGs

The Limma package was used to test expression differences between CESC tumors and normal cervix samples in TCGA. Differentially expressed genes (DEGs) were defined using |log2FC| > 1 together with adjusted P < 0.05. The intersection between DEGs and the curated lymphangiogenesis-related gene set yielded differentially expressed lymphangiogenesis-related genes (DELYMRGs). We first filtered out patients with incomplete survival records and those with overall survival <30 days. Each DELYMRG was subsequently evaluated by single-gene Cox regression in the survival package, and significant genes (P < 0.05) were carried forward.

### 2.3 Construction and validation of the prognostic model

To obtain a compact prognostic signature, we fit an L1-regularized Cox model to the prognostic DELYMRGs using the glmnet package. The regularization strength (λ) was tuned by k-fold cross-validation, and genes with non-zero coefficients under the optimal solution were retained to derive a parsimonious predictor set. We constructed the final signature by fitting a multivariable Cox model (survival) to the features retained after least absolute shrinkage and selection operator (LASSO) selection. The linear predictor from this model was used to calculate individual risk scores ( $\text{RiskScore}=\Sigma \;\left(\beta i\times \text{Expi}\right)$ ; βi, estimated coefficient; Expi, gene expression). The cohort was subsequently split at the median score to define high-versus low-risk groups. Kaplan-Meier analysis was used to test whether the risk score stratified overall survival. We then quantified time-dependent receiver operating characteristic (ROC) curves using timeROC (AUC at 1, 3, and 5 years). Finally, risk-score rankings and survival status were plotted to provide a visual depiction of group separation. The prognostic performance of the model was further evaluated in the external validation cohort and/or the full TCGA-CESC cohort by repeating Kaplan–Meier and time-dependent ROC analyses, along with corresponding RiskScore distribution and survival status visualizations. Kaplan-Meier analyses were run on a per-gene basis in the training cohort. We further visualized expression of the signature genes using boxplots across tumor and normal samples, and across high- and low-risk strata.

### 2.4 Molecular subtype identification based on the LYMRGs

TCGA-CESC samples were grouped according to the expression profiles of the six signature genes. Subtypes were derived using NMF implemented in the NMF package, with the number of clusters determined by the optimal factorization rank, resulting in two molecular subtypes. We compared overall survival between Cluster 1 and Cluster 2 using Kaplan-Meier analysis followed by log-rank tests. Differential expression between the two clusters was evaluated with limma, and genes meeting |log_2_FC| > 1 and adjusted P < 0.05 were retained. To interpret the biological functions of subtype-associated DEGs, we carried out Gene Ontology (GO) and Kyoto Encyclopedia of Genes and Genomes (KEGG) over-representation analyses using clusterProfiler. We then evaluated the prognostic implication of these subtype DEGs by performing gene-level survival analyses to prioritize outcome-related candidates.

### 2.5 Immune microenvironment analyses

Single-sample gene set enrichment analysis (ssGSEA) was used to score immune-cell infiltration and immune functional signatures in each sample. These scores were then compared between molecular subtypes to delineate subtype-specific immune patterns. Differences in ssGSEA scores between subtypes were compared using non-parametric tests. We quantified immune and stromal content with ESTIMATE (estimate package) by deriving ImmuneScore, StromalScore, ESTIMATEScore, and tumor purity for each sample, followed by between-subtype comparisons. In parallel, CIBERSORT deconvolution was used to estimate fractions of 22 immune cell types, and differences in cell-type abundance were evaluated between the high- and low-risk groups.

### 2.6 Functional enrichment analyses

Pathway enrichment was assessed by Gene Set Enrichment Analysis (GSEA) using the desktop software (v4.3.3) and KEGG gene sets. Enrichment patterns were compared between the high- and low-RiskScore strata. Genes were ranked according to their differential expression between the two groups, and enrichment was evaluated using permutation-based testing under standard settings. To capture transcriptional changes linked to the RiskScore, we performed limma-based differential expression testing between the high- and low-risk strata (|log_2_FC| > 1; adjusted P < 0.05). The resulting gene list was subsequently annotated by GO and KEGG enrichment to summarize risk-related biological programs.

### 2.7 Clinical utility and independent prognostic evaluation of the signature

To assess the clinical applicability of the signature, we related the RiskScore to key clinicopathological variables and tested whether the score differed across clinical strata. Differences in RiskScore across clinical subgroups were assessed using appropriate statistical tests depending on variable type. For each marker, patients were split at the median expression level to construct high/low expression strata, and Kaplan–Meier curves were used to evaluate survival separation. To test whether the RiskScore provides prognostic information beyond clinical variables, we fitted unadjusted and covariate-adjusted Cox regression models incorporating the RiskScore and relevant clinical factors. A RiskScore-based nomogram incorporating clinical covariates was developed with the rms package. Calibration plots and decision curve analysis (DCA) were used to evaluate predictive accuracy and clinical net benefit across threshold probabilities, respectively.

### 2.8 Immune microenvironment analyses

Immune profiles were scored by ssGSEA across 28 immune cell populations together with immune functional signatures, yielding an enrichment score for each sample. Group-wise differences (high vs. low RiskScore) were visualized with boxplots. Tumor purity–related microenvironment metrics, including immune and stromal signals, were further derived using ESTIMATE (estimate package). For each sample, we obtained ESTIMATE scores (ImmuneScore, StromalScore, and ESTIMATEScore) together with inferred tumor purity, followed by high/low-risk comparisons. We complemented this with LM22-based CIBERSORT deconvolution to profile 22 immune-cell fractions and test group-level differences. Checkpoint-gene expression was also contrasted between risk strata. Associations between immune-cell fractions and signature-gene expression were quantified by Pearson correlation and visualized as a heatmap. To infer potential response to immune checkpoint blockade, we calculated Tumor Immune Dysfunction and Exclusion (TIDE) scores and compared their distributions between the high- and low-risk groups using violin plots. Immunophenoscore (IPS) values for TCGA-CESC were retrieved from The Cancer Immunome Atlas (TCIA) and similarly contrasted across risk strata. In addition, tumor immune subtypes (C1–C6; C1 wound healing, C2 IFN-γ dominant, C3 inflammatory, C4 lymphocyte depleted, C5 immunologically quiet, and C6 TGF-β dominant) were assigned based on published immunogenomic annotations (Thorsson et al., 2018).

### 2.9 Drug sensitivity prediction and ceRNA network construction

Chemotherapy response was estimated from transcriptomic profiles using pRRophetic, which outputs predicted the half-maximal inhibitory concentration (IC50) values for commonly used anticancer agents at the individual-sample level. Predicted IC50 distributions were then contrasted between the high- and low-risk groups to highlight compounds with differential sensitivity. To further probe gene–drug links, we leveraged the CellMiner resource and quantified associations between signature-gene expression and drug activity metrics using Spearman correlation, prioritizing candidate gene–drug pairs. For competing endogenous RNA (ceRNA) network construction, experimentally supported miRNA–target interactions were collected from miRTarBase (https://mirtarbase.cuhk.edu.cn/). To ensure the reliability of the ceRNA network, we prioritized miRNA–mRNA interactions supported by strong experimental evidence, thereby reducing the reliance on purely computational predictions. MiRNAs targeting the signature genes were extracted based on the curated interaction records. LncRNA–miRNA interaction data were downloaded from ENCORI (https://rnasysu.com/encori/), and lncRNAs with strong supporting evidence (clipExpNum >30) were retained. The integrated lncRNA–miRNA–mRNA regulatory network was visualized using Cytoscape (v3.10.2).

### 2.10 Statistical analysis

All analyses were carried out in R (v4.4.2) with publicly available packages. Two-group comparisons used the Wilcoxon rank-sum test. Overall survival was evaluated with Kaplan-Meier analysis and log-rank testing. Time-dependent discrimination was quantified by AUCs at prespecified time points using the timeROC package. Associations between continuous variables were examined by correlation analysis, applying Pearson’s method for approximately normally distributed linear relationships and Spearman’s rank correlation otherwise. Figures were produced mainly with ggplot2. Median values were used as cutoffs for dichotomization in grouping analyses (e.g., RiskScore- or gene expression–based stratification). A two-sided P value <0.05 was considered statistically significant. Significance levels were denoted as follows: ****, P < 0.0001; ***, 0.0001 < P < 0.001; **, 0.001 < P < 0.01; *, 0.01 < P < 0.05; ns, P > 0.05.

### 2.11 Cell culture

The human cervical carcinoma cell line HeLa, SiHa and the human cervical epithelial cell line H8 were used for *in vitro* validation. Cells were maintained under standard sterile conditions in Dulbecco’s modified Eagle’s medium (DMEM, high glucose) supplemented with 10% fetal bovine serum (FBS) and 1% penicillin–streptomycin in a humidified incubator at 37 °C with 5% CO_2_. Culture medium was refreshed every 2–3 days, and cells were passaged at approximately 70%–80% confluence using 0.25% trypsin–EDTA. Cells in the logarithmic growth phase were used for subsequent RNA extraction and quantitative PCR assays.

### 2.12 RNA extraction and real-time quantitative PCR (RT-qPCR)

Total RNA was extracted from HeLa, SiHa and H8 cells using the RNA Eazy Fast Cell Kit (TIANGEN Biotech Co., Beijing, China) according to the manufacturer’s instructions. RNA concentration and purity were assessed using a NanoDrop 2000 spectrophotometer (Thermo Fisher Scientific, United States of America). Subsequently, 2 μg of total RNA was reverse transcribed into complementary DNA (cDNA) using the FastKing RT Kit (TIANGEN Biotech Co., Beijing, China). Real-time quantitative PCR was performed with SuperReal PreMix Plus (TIANGEN Biotech Co., Beijing, China) on an Applied Biosystems quantitative real-time PCR system following the manufacturer’s protocols. Relative mRNA expression levels of the target genes were normalized to an internal reference gene and calculated using the 2^−ΔΔCT^ method. Primer sequences used in this study are listed in Table 1. All reactions were performed with technical replicates, and independent experiments were repeated as appropriate.

**TABLE 1:** Sequences of primers for qRT-PCR.

Gene name	Primer sequences (5′-3′)
EFNA1	Forward: AAA​CCC​ATC​CAC​CAG​CAT​GA
	Reverse: GCC​TGA​GGA​CTG​TGA​GTG​ATT
FASN	Forward: GCA​AGC​TGA​AGG​ACC​TGT​CT
	Reverse: AAT​CTG​GGT​TGA​TGC​CTC​CG
NRP1	Forward: TGT​GAA​GTG​GAA​GCC​CCT​AC
	Reverse: CAC​CTG​TGA​GCT​GGA​AGT​CA
ZIC2	Forward: AAA​GGA​CCC​ACA​CAG​GGG​AGA
	Reverse: GAC​GTG​CAT​GTG​CTT​CTT​CCT
IL-1B	Forward: CCA​AAC​CTC​TTC​GAG​GCA​CA
	Reverse: GGC​TGC​TTC​AGA​CAC​TTG​AG
FOXP3	Forward: CAG​GAA​GGA​CAG​CAC​CCT​TT
	Reverse: GAC​ACC​ATT​TGC​CAG​CAG​TG

### 2.13 Cell transfection and dual-luciferase reporter assay

SiRNA targeting NEAT1 (si-NEAT1), miR-124–3p mimics, and their negative controls (si-NC, mimics NC) were synthesized. Cells were transfected using Lipofectamine 3,000 (Invitrogen). For the luciferase assay, wild-type (WT) and mutant (MUT) sequences of EFNA1 3′UTR and NEAT1 were cloned into the pmirGLO vector. SiHa cells were co-transfected with reporter plasmids and miRNA mimics. Luciferase activity was measured 48 h post-transfection using the Dual-Luciferase Reporter Assay System.

### 2.14 Western blot (WB) analysis

Total protein was extracted from SiHa cells using RIPA buffer. Protein samples were separated by SDS-PAGE and transferred to PVDF membranes. Membranes were incubated with primary antibodies against EFNA1 (1:1,000) and GAPDH (1:5,000) overnight at 4 °C, followed by secondary antibodies. Protein bands were visualized using ECL reagents and quantified using ImageJ software.

## 3 Results

### 3.1 Construction and validation of a lymphangiogenesis-related prognostic risk signature in CESC

**FIGURE 1:**
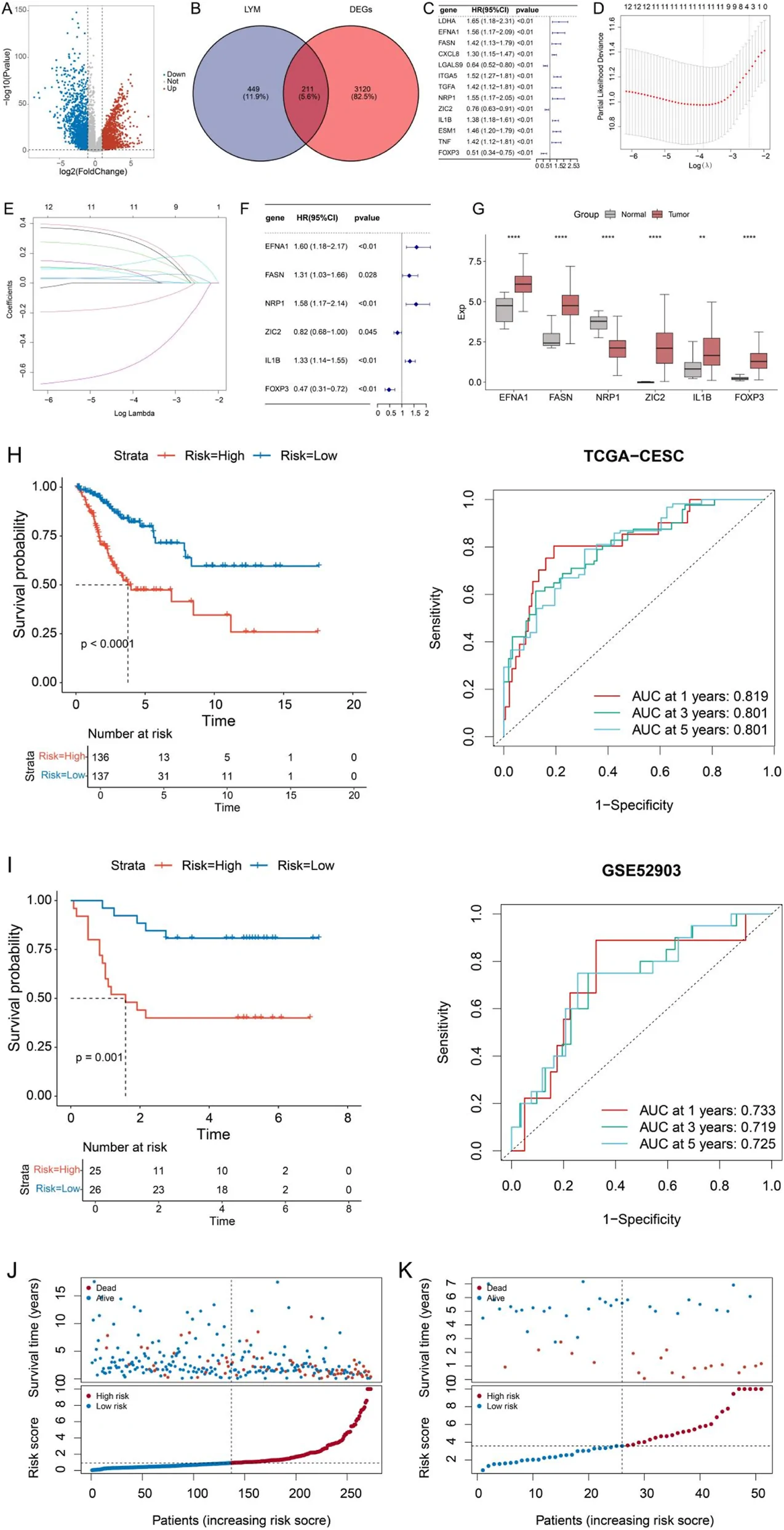
Identification of prognostic LYMRGs and construction of a six-gene risk signature in CESC. **(A)** Volcano plot of DEGs between CESC tumors and normal cervix samples. **(B)** Venn diagram showing the intersection of DEGs and LYMRGs, yielding 211 DELYMRGs. **(C)** Forest plot of univariate Cox regression analysis of DELYMRGs identifying prognosis-associated candidates (P < 0.005). **(D)** Cross-validation curve for the LASSO Cox model used to determine the optimal penalty parameter (λ). **(E)** LASSO coefficient profiles of candidate genes across log(λ). **(F)** Forest plot of multivariable Cox regression defining the final six-gene signature. **(G)** Expression distribution of the six signature genes in tumor versus normal samples. **(H)** Kaplan–Meier survival curves and time-dependent ROC curves (1-, 3-, and 5-year AUCs) for the high- and low-risk groups in the TCGA-CESC training cohort. **(I)** Kaplan–Meier survival curves and time-dependent ROC curves in the independent GEO validation cohort (GSE52903). **(J)** RiskScore distribution and survival status in the TCGA-CESC cohort. **(K)** RiskScore distribution and survival status in the GSE52903 cohort using the same stratification strategy.

We first profiled transcriptomic differences between cervical cancer and normal cervix tissues, yielding 3,331 DEGs (Figure 1A). Overlaying these DEGs with the lymphangiogenesis-related gene set resulted in 211 DELYMRGs (Figure 1B). Univariable Cox proportional hazards modeling further narrowed the list to 13 survival-associated DELYMRGs (Figure 1C). To derive a parsimonious prognostic signature, we then applied LASSO-penalized Cox regression and retained 11 genes at the optimal penalty parameter (Figures 1D,E; Supplementary Table S2). These genes were subsequently entered into a multivariable Cox regression model, resulting in a six-gene prognostic signature comprising EFNA1, FASN, NRP1, ZIC2, IL1B, and FOXP3 (Figure 1F). Across the six-gene panel, expression levels differed markedly between tumor and normal cervix tissues. Specifically, EFNA1, FASN, ZIC2, IL1B, and FOXP3 were expressed at higher levels in tumor tissues compared with normal controls, whereas NRP1 showed relatively higher expression in normal samples (Figure 1G). Patients were split at the median RiskScore to define two risk strata in the TCGA training cohort. Survival curves indicated significant OS separation, with the high-risk group exhibiting poorer outcomes (P < 0.0001; Figure 1H, left). Time-dependent ROC curves confirmed predictive performance, with AUCs of 0.819 (1 year), 0.801 (3 years), and 0.801 (5 years) (Figure 1H, right). We next assessed model generalizability in an external GEO dataset (GSE52903). The distribution of RiskScore and survival status in the training cohort showed an increased death event density with rising RiskScore (Figure 1J). External validation was conducted in the independent GEO cohort GSE52903. Consistently, high-risk patients exhibited significantly worse survival (P = 0.001; Figure 1I, left), and the model maintained stable discriminatory performance with AUCs of 0.733, 0.719, and 0.725 at 1, 3, and 5 years, respectively (Figure 1I, right). RiskScore distribution and survival status plots in the validation set recapitulated the trend observed in the training cohort, with unfavorable outcomes concentrated in the high-risk stratum (Figure 1K). Exploration of ALKBH5-related transcriptional associations with signature genes given prior evidence implicating ALKBH5-mediated m6A demethylation in lymphangiogenesis-related processes, we further explored transcriptional correlations between ALKBH5 and the six signature genes. In the overall cohort, ALKBH5 expression was significantly correlated with FOXP3 and IL1B (Supplementary Figure S1). Notably, risk-stratified analyses suggested context dependence: significant associations were observed in the low-risk group (including EFNA1, ZIC2, and FOXP3), whereas no significant correlations were detected between ALKBH5 and the signature genes in the high-risk group (Supplementary Table S3). These findings suggest a potential risk-dependent rewiring of ALKBH5-linked regulatory programs in CESC.

### 3.2 Molecular subtyping based on the six-gene lymphangiogenesis-related signature

**FIGURE 2:**
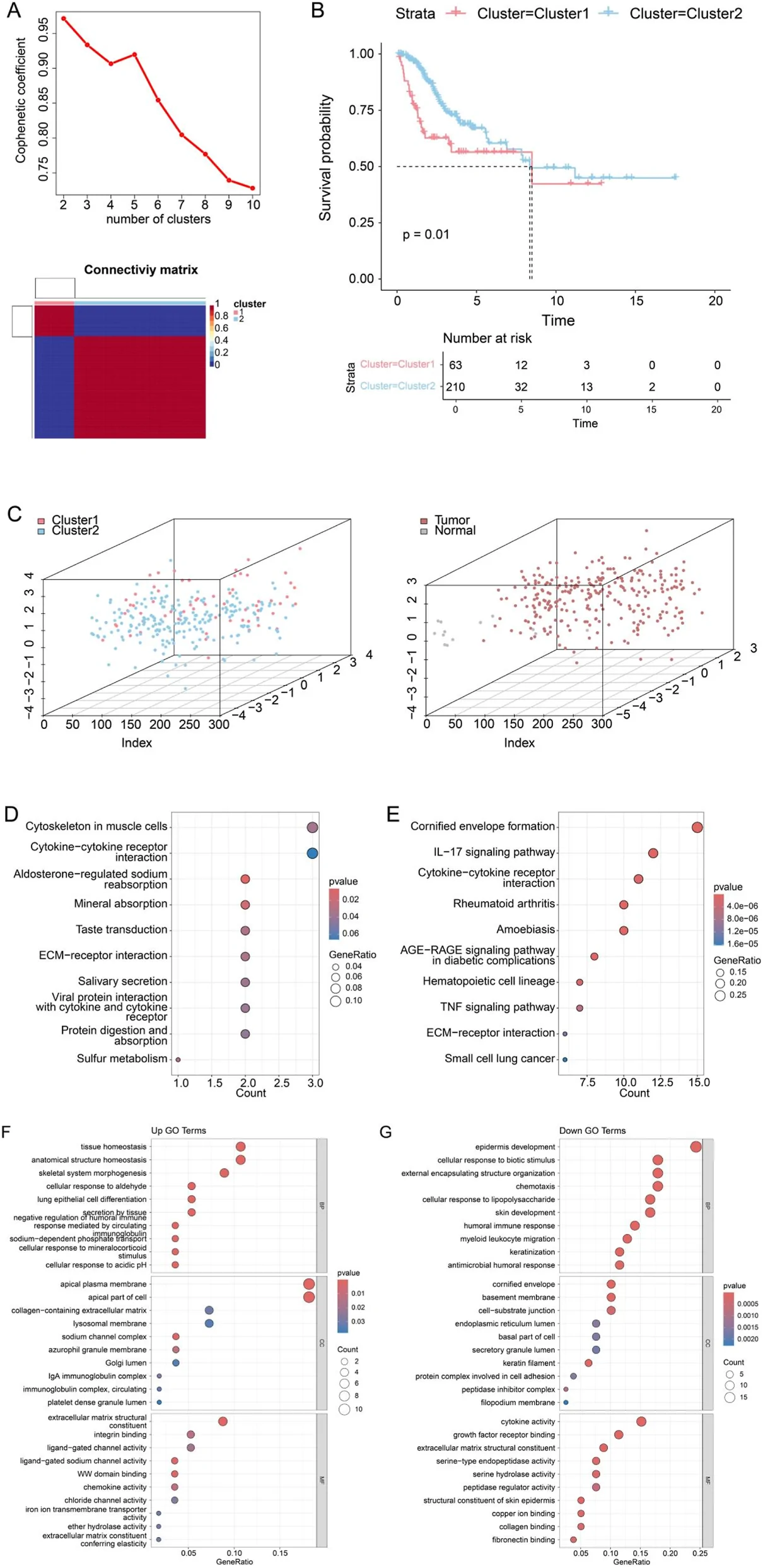
NMF-based molecular subtypes defined by the six-gene lymphangiogenesis-related signature and their functional characteristics in TCGA-CESC. **(A)** Determination of the optimal number of clusters for NMF. **(B)** Kaplan–Meier survival curves comparing overall survival between Cluster1 and Cluster2. **(C)** PCA based on the expression of the six signature genes, showing separation of the two molecular subtypes (left) and discrimination between tumor and normal samples (right). **(D,E)** KEGG pathway enrichment dot plots for subtype-associated DEGs between Cluster1 and Cluster2. **(F)** GO enrichment results for genes upregulated between subtypes (Up GO Terms), displayed across BP, CC, and MF categories. **(G)** GO enrichment results for genes downregulated between subtypes (Down GO Terms), displayed across BP, CC, and MF categories.

To investigate molecular heterogeneity associated with the lymphangiogenesis-related prognostic signature, we performed NMF clustering on TCGA-CESC tumors using the expression profiles of the six signature genes. Evaluation of the NMF runs indicated that k = 2 provided the most coherent partitioning, supported by the cophenetic profile and the corresponding consensus/connectivity structure (Figure 2A). Survival analysis further highlighted clinical heterogeneity between subtypes, with worse outcomes in Cluster 1 (P < 0.01; Figure 2B). PCA of the six-gene expression matrix recapitulated this separation and additionally differentiated tumor from normal tissues (Figure 2C). To explore subtype-associated transcriptional programs, differential expression analysis was performed between Cluster1 and Cluster2, yielding 140 subtype-related DEGs under the predefined thresholds. Functional interpretation of these DEGs revealed distinct pathway-level patterns. KEGG enrichment dot plots highlighted subtype-associated pathways enriched among DEG subsets, including aldosterone-regulated sodium reabsorption and mineral absorption (Figure 2D) as well as cornified envelope formation, IL-17 signaling pathway and cytokine–cytokine receptor interaction (Figure 2E). Consistently, GO enrichment analyses showed that genes upregulated in one subtype were associated with homeostatic (e.g., tissue homeostasis and anatomical structure homeostasis) and immune-related processes and membrane/extracellular matrix components (e.g., extracellular matrix structural constituent, apical plasma membrane, and apical part of cell) (Figure 2F), whereas genes downregulated in the other subtype were enriched for epidermis/skin development, chemotaxis and immune response terms, and extracellular matrix/structural and binding functions (Figure 2G).

### 3.3 Immune landscape differences between the two molecular subtypes

**FIGURE 3:**
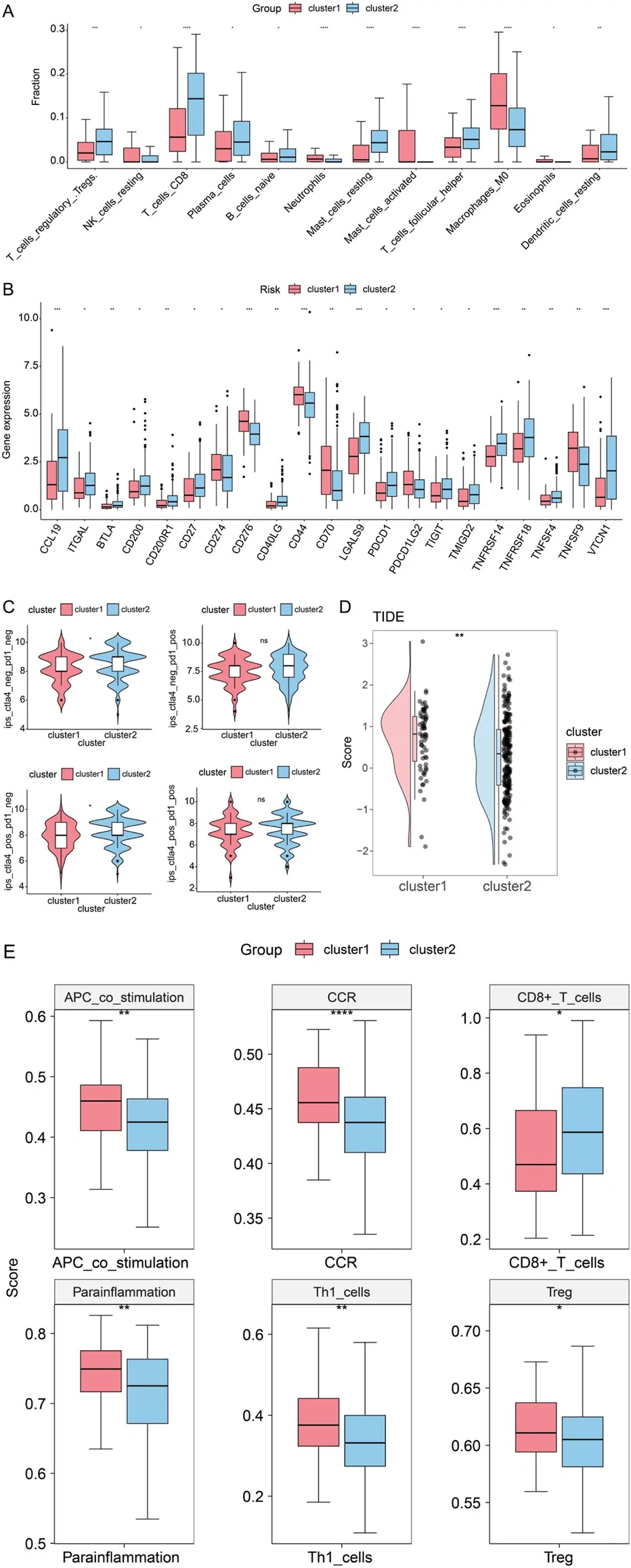
Immune landscape and immunotherapy-related features of the two NMF-defined molecular subtypes in TCGA-CESC. **(A)** Comparison of immune cell infiltration between Cluster1 and Cluster2 estimated by CIBERSORT. **(B)** Differential expression of immune checkpoint–related and immunoregulatory genes between the two subtypes. **(C)** IPS distributions from TCIA under four immune checkpoint blockade scenarios for Cluster1 versus Cluster2. **(D)** TIDE scores comparing Cluster1 and Cluster2. **(E)** SsGSEA-based immune signature scores (representative immune functions and immune cell–related signatures) compared between Cluster1 and Cluster2.

To characterize immune heterogeneity across the NMF-defined subtypes, we first compared immune cell infiltration profiles inferred by CIBERSORT. Immune deconvolution profiles differed markedly between the two molecular subtypes (Figure 3A). In particular, Cluster 2 displayed higher estimated proportions of regulatory T cells (Tregs), CD8^+^ T cells, plasma cells, naïve B cells, resting mast cells, T follicular helper cells, and resting dendritic cells compared with Cluster 1. By comparison, Cluster 1 was characterized by increased proportions of resting NK cells, neutrophils, activated mast cells, M0 macrophages, and eosinophils, highlighting divergent immune infiltration patterns across subtypes. We subsequently assessed immune checkpoint and other immunotherapy-associated genes, and identified several markers with subtype-specific expression (Figure 3B). Cluster2 displayed higher expression of multiple immune activation/inhibitory–associated genes, including CCL19, CD27, and CD274 (PD-L1). Conversely, Cluster1 showed higher expression of several genes such as CD276, CD44, CD70, PDCD1LG2, and TNFSF9, suggesting divergent checkpoint signaling contexts between the two clusters. To estimate potential responsivenees to immune checkpoint blockade, IPS values were compared between subtypes under four therapeutic scenarios (Figure 3C). IPS comparisons indicated a subtype effect restricted to PD-1–negative scenarios: Cluster 2 had higher IPS in CTLA4−/PD1− and CTLA4+/PD1−, but not in the PD-1–positive settings (Figure 3C). TIDE analysis likewise supported subtype divergence, as Cluster 1 exhibited higher TIDE scores than Cluster 2 (Figure 3D). Finally, ssGSEA-based immune signature scoring demonstrated broad subtype-dependent differences across immune-related functional programs and representative immune cell signatures (Figure 3E). Cluster1 had higher scores for APC co-stimulation, chemokine receptor activity (CCR), parainflammation, Th1 cell signatures, and Treg-related signatures, whereas Cluster2 showed a higher CD8^+^ T-cell signature score. Collectively, these findings indicate that the two molecular subtypes defined by the lymphangiogenesis-related signature are associated with distinct immune infiltration landscapes and immunotherapy-relevant phenotypes.

### 3.4 Independent prognostic value of the risk signature and nomogram construction

**FIGURE 4:**
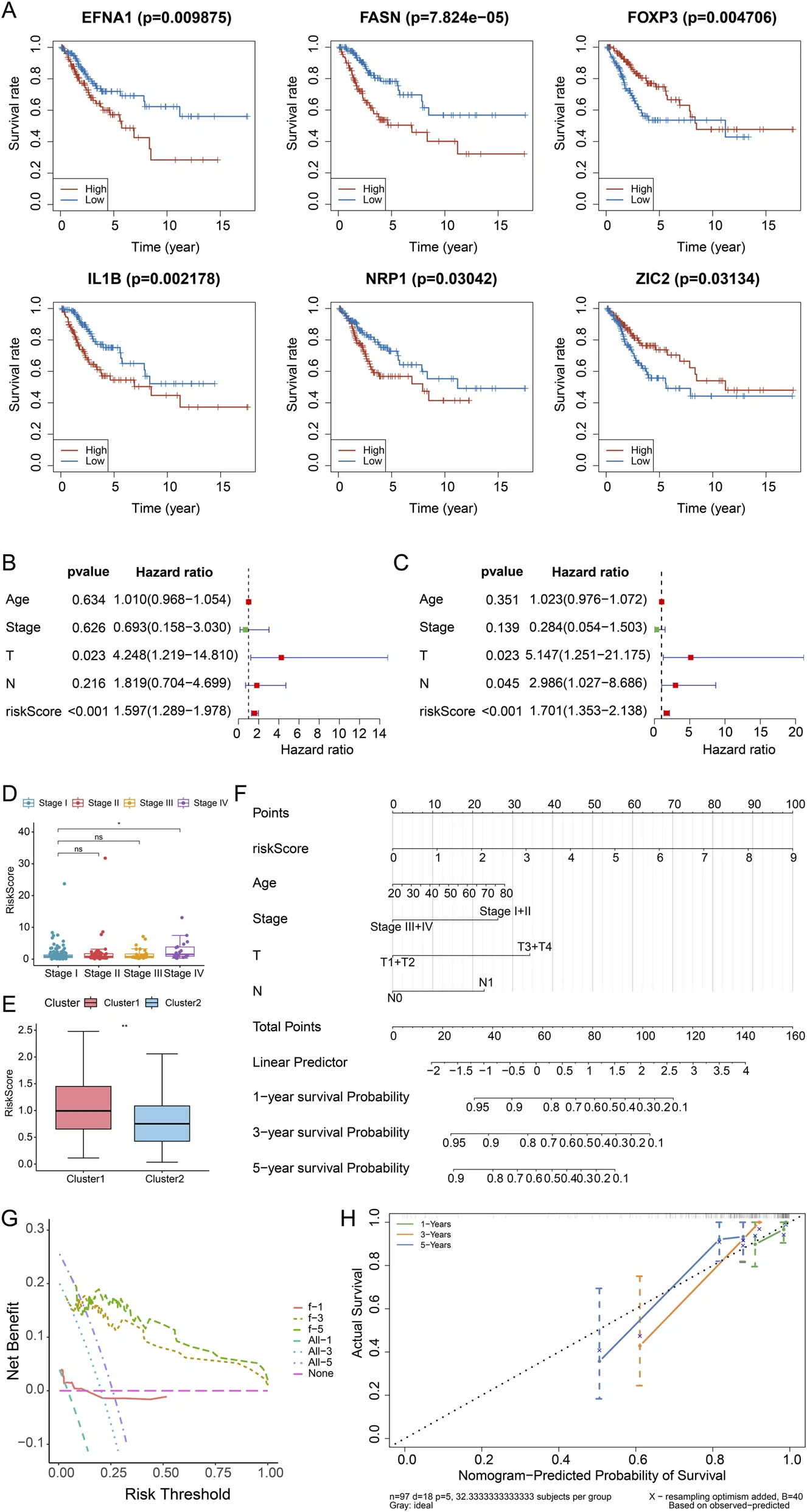
Independent prognostic evaluation of the six-gene signature and development of a nomogram in TCGA-CESC. **(A)** Kaplan–Meier survival curves for the six signature genes (EFNA1, FASN, FOXP3, IL1B, NRP1, and ZIC2) stratified by high versus low expression. **(B)** Univariate Cox regression analysis of clinicopathological variables and RiskScore. **(C)** Multivariable Cox regression analysis demonstrating the independent prognostic value of RiskScore after adjustment for clinical covariates. **(D)** Comparison of RiskScore between FIGO stages I–II and FIGO stages III–IV. **(E)** Comparison of RiskScore between HPV16-positive cases and other subtypes. **(F)** Comparison of RiskScore between the two NMF-defined molecular subtypes (Cluster1 vs. Cluster2). **(G)** Nomogram integrating RiskScore and clinical variables to predict 1-, 3-, and 5-year overall survival. **(H)** DCA assessing the net clinical benefit of the nomogram across a range of threshold probabilities. **(I)** Calibration plots for the nomogram showing agreement between predicted and observed 1-, 3-, and 5-year survival probabilities. **(J)** Time-dependent receiver operating characteristic (ROC) curves for the nomogram predicting 1-, 3-, and 5-year overall survival.

For each signature gene, we assessed survival associations by Kaplan-Meier analysis after dichotomizing patients at the median expression level (Figure 4A). High expression of EFNA1 (P = 0.0099), FASN (P = 7.82 × 10^−5^), IL1B (P = 0.0022), and NRP1 (P = 0.030) was associated with significantly worse overall survival, whereas high expression of FOXP3 (P = 0.0047) and ZIC2 (P = 0.031) was linked to improved survival, consistent with their respective roles in the model. We next assessed whether the RiskScore provided prognostic information beyond routine clinicopathological variables (Figures 4B,C). In both univariate and multivariable Cox analyses, the RiskScore remained a significant independent prognostic factor (HR = 1.701, 95% CI: 1.353–2.138; P < 0.001). Notably, N stage (HR = 2.986, P = 0.045) also retained significance, supporting the clinical relevance of incorporating lymphangiogenesis-related molecular features into traditional risk assessment. RiskScore distributions across clinical strata further substantiated its prognostic utility. The RiskScore was significantly higher in patients with advanced FIGO stages (III–IV) than in those with early stages (I–II) (P < 0.05, Figure 4D) and was markedly lower in HPV16-positive cases compared to other high-risk HPV subtypes (P < 0.05, Figure 4E). While associations with LNM and LVSI status were not statistically significant (Supplementary Figure S2)—likely due to the high rate of missing data in the TCGA records—the overall trends suggest that the RiskScore serves as a molecular indicator of metastatic potential that may precede detectable structural changes. Additionally, Cluster 1 exhibited a significantly higher RiskScore than Cluster 2 (Figure 4F). To facilitate individualized survival prediction, a nomogram integrating RiskScore with clinical variables was constructed (Figure 4G). The nomogram exhibited exceptional discriminative performance, with 1-, 3-, and 5-year AUCs of 0.806, 0.941, and 0.849, respectively (Figure 4J). This superior predictive accuracy over individual clinical parameters suggests that the model provides substantial additional prognostic benefits. DCA demonstrated a favorable net clinical benefit across a wide range of threshold probabilities (Figure 4H), and calibration plots showed good agreement between predicted and observed 1-, 3-, and 5-year survival probabilities (Figure 4I). Collectively, these results support the six-gene RiskScore as a robust independent prognostic indicator with significant clinical utility.

### 3.5 Immune infiltration and immunogenomic features associated with the RiskScore

**FIGURE 5:**
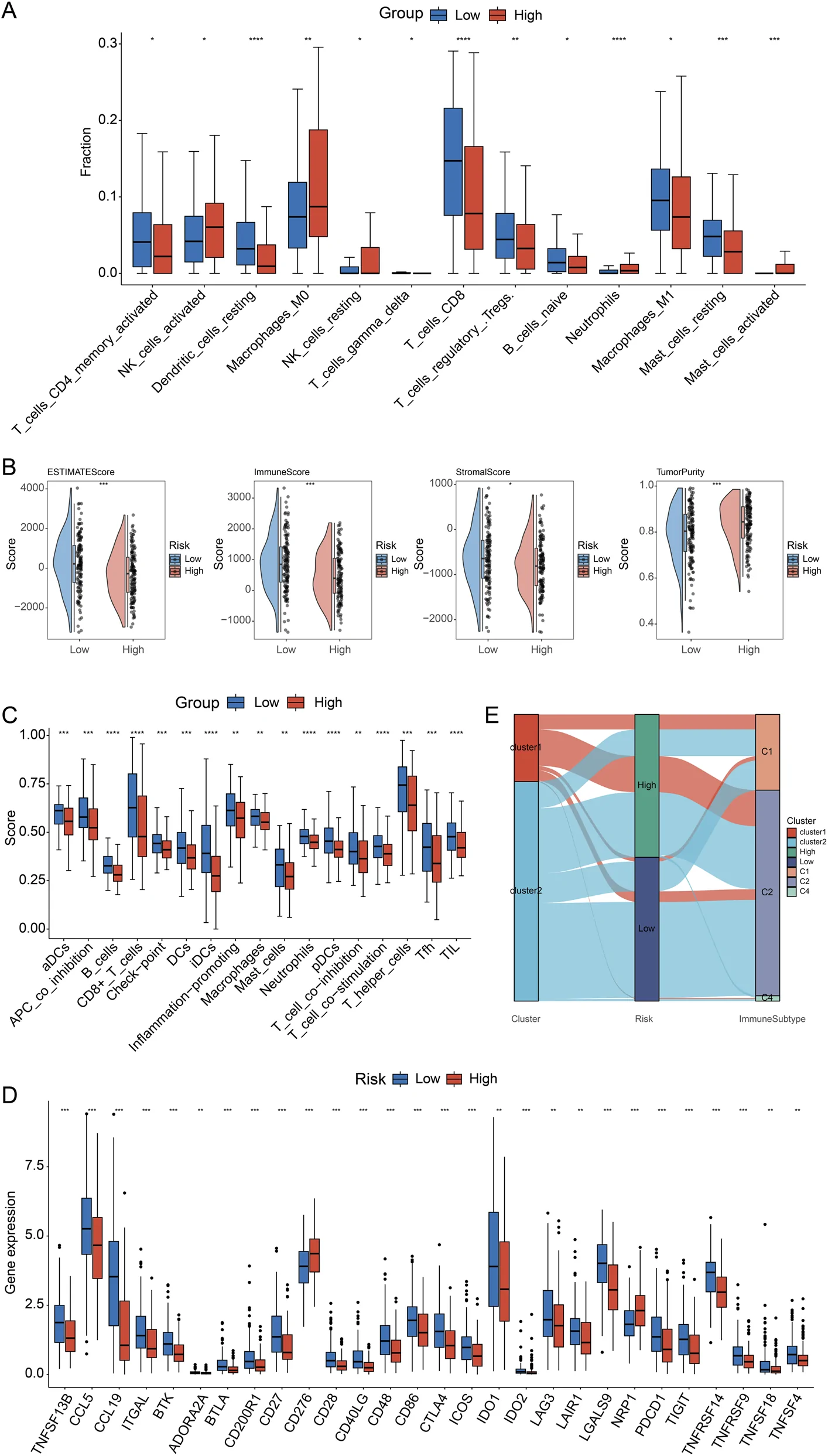
Immune infiltration landscape and immunogenomic features associated with the RiskScore in CESC. **(A)** Comparison of immune cell infiltration between low- and high-risk groups estimated by CIBERSORT. **(B)** ESTIMATE-derived tumor microenvironment scores (ESTIMATEScore, ImmuneScore, StromalScore) and tumor purity compared between low- and high-risk groups. **(C)** SsGSEA-based immune signature scores (immune functions and immune cell–related signatures) compared between low- and high-risk groups. **(D)** Differential expression of immune checkpoint–related and immunoregulatory genes between low- and high-risk groups. **(E)** Sankey diagram illustrating the relationships among molecular subtype (Cluster1/Cluster2), RiskScore group (High/Low), and TCGA pan-cancer immune subtypes (C1–C6).

CIBERSORT-based deconvolution revealed significant differences in immune cell composition between the low- and high-risk groups (Figure 5A). Compared with the high-risk group, the low-risk group exhibited higher fractions of activated CD4 memory T cells, resting dendritic cells, activated NK cells, CD8^+^ T cells, Tregs, naive B cells, M1 macrophages, and resting mast cells. In contrast, the high-risk group showed relatively increased infiltration of activated NK cells, M0 macrophages, resting NK cells, and activated mast cells. Collectively, these patterns suggest a more “T cell–inflamed” microenvironment in the low-risk group, whereas the high-risk group is characterized by enrichment of innate/stromal-associated components and macrophage M0 predominance. Consistent with these findings, ESTIMATE analysis demonstrated that overall microenvironmental content differed substantially between risk strata (Figure 5B). The low-risk group had significantly higher ImmuneScore, StromalScore, and the composite ESTIMATEScore, whereas tumor purity was significantly higher in the high-risk group, indicating reduced non-tumor (immune/stromal) infiltration in high-risk tumors. Using ssGSEA, we further profiled immune-related functional programs and immune cell–associated signatures (Figure 5C). Across a broad panel of signatures—including antigen-presenting cell–related activity (APC co-stimulation/DC-related signatures), checkpoint-related signatures, and multiple T-cell–associated programs (e.g., T helper, Tfh, Th1, and TIL signatures)—the low-risk group consistently showed higher enrichment scores than the high-risk group, supporting an immune-activated phenotype in low-risk tumors. We next examined the expression of immune checkpoint–related and immunoregulatory genes (Figure 5D). Most T cell–inflamed and costimulatory/inhibitory molecules (including PDCD1, CTLA4, ICOS, LAG3, TIGIT, as well as chemokine/immune trafficking–related genes such as CCL5 and CCL19) were expressed at higher levels in the low-risk group, in line with its higher immune infiltration. In contrast, CD276 and NRP1 were relatively upregulated in the high-risk group, suggesting that high-risk tumors may preferentially engage alternative immune-evasion or stromal/vascular-associated regulatory axes. Finally, a Sankey diagram integrating molecular subtype, RiskScore group, and immunogenomic immune subtype assignments indicated a non-random relationship among these classifications (Figure 5E). Cluster2 was predominantly associated with the low-risk group and was enriched for the C2 (IFN-γ–dominant) immune subtype, whereas Cluster1 contributed a larger proportion of high-risk cases and showed a higher representation of C1 (wound healing) features, with C4 (lymphocyte depleted) appearing in a minority of samples. These results suggest that the lymphangiogenesis-related stratification captures coordinated variation in tumor immune contexture and immunogenomic subtype.

### 3.6 Functional enrichment analyses between high- and low-risk groups

**FIGURE 6:**
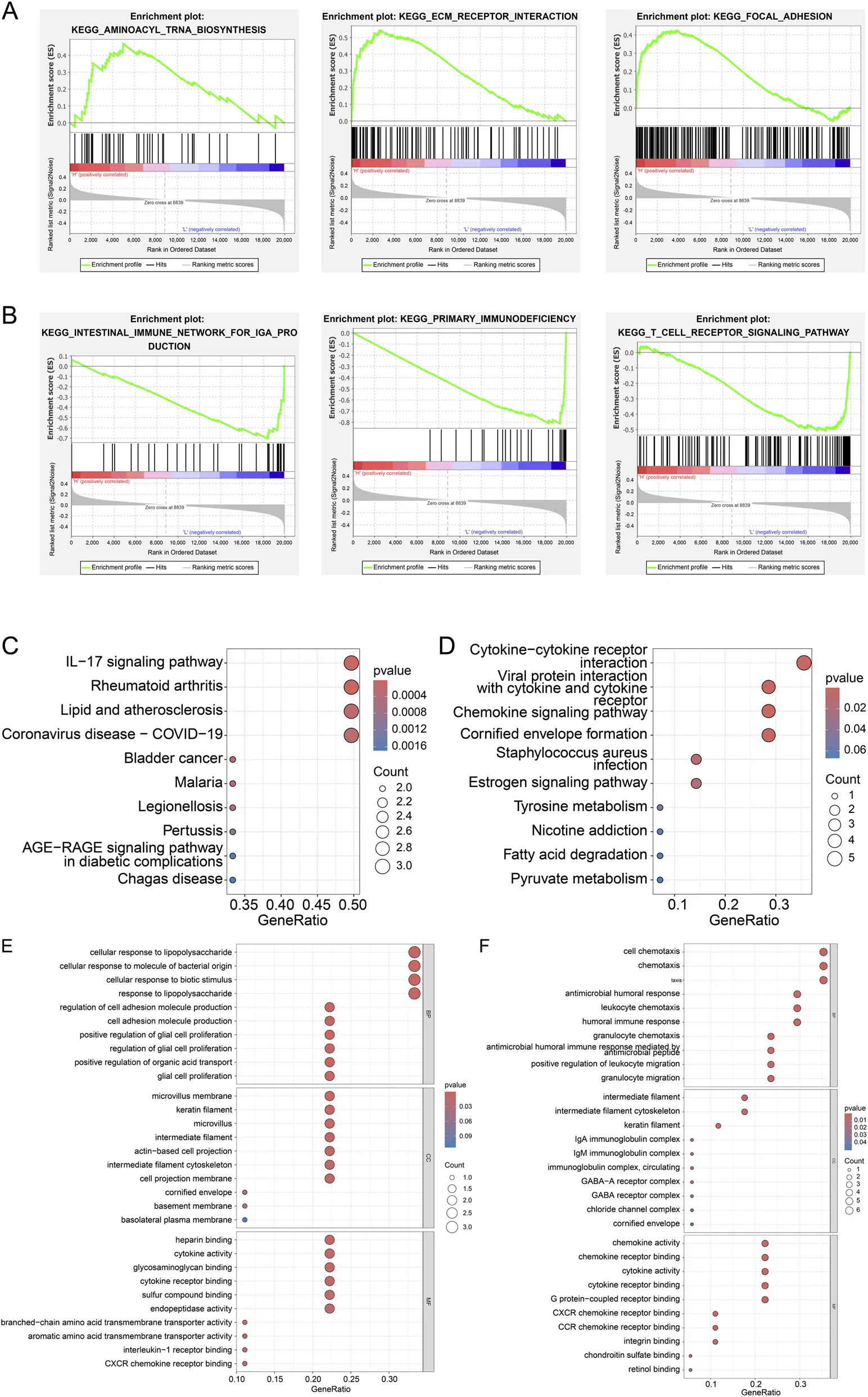
Pathway enrichment analyses associated with the RiskScore in TCGA-CESC. **(A,B)** GSEA of KEGG pathways in the high-risk group **(A)** and low-risk group **(B). (C,D)** KEGG pathway enrichment dot plots based on DEGs between high- and low-risk groups. **(E,F)** GO enrichment results for risk-associated DEGs, displayed across biological process (BP), cellular component (CC), and molecular function (MF) categories.

Gene set enrichment analysis (GSEA) based on the KEGG collection revealed distinct pathway-level programs associated with risk strata. In the high-risk group, enrichment plots highlighted significant activation of pathways related to extracellular matrix remodeling and cell adhesion/motility, including ECM–receptor interaction and focal adhesion, along with aminoacyl-tRNA biosynthesis (Figure 6A). In contrast, the low-risk group was significantly enriched for immune-related pathways, such as intestinal immune network for IgA production, primary immunodeficiency, and T cell receptor signaling (Figure 6B), consistent with an immune-inflamed phenotype in low-risk tumors. To further delineate transcriptional differences, differential expression analysis between the two risk groups (|log_2_FC| > 1, adjusted P < 0.05) identified 27 risk-associated genes, including 9 upregulated and 18 downregulated genes. KEGG enrichment of these DEGs showed prominent immune/inflammatory signaling and cytokine-related pathways, including IL-17 signaling pathway, cytokine–cytokine receptor interaction, and chemokine signaling pathway, as well as additional metabolic and hormonal signaling modules such as estrogen signaling (Figures 6C,D). GO analyses further supported these functional patterns, with enriched biological processes and molecular functions associated with immune responses (e.g., chemotaxis/leukocyte migration and cytokine/chemokine activity) and structural components (e.g., intermediate filament/keratin-related terms and extracellular/binding-related functions) (Figures 6E,F). Collectively, these enrichment results suggest that high RiskScore tumors preferentially engage ECM–adhesion programs linked to invasion, whereas low RiskScore tumors retain stronger immune signaling signatures.

### 3.7 Drug sensitivity prediction

**FIGURE 7:**
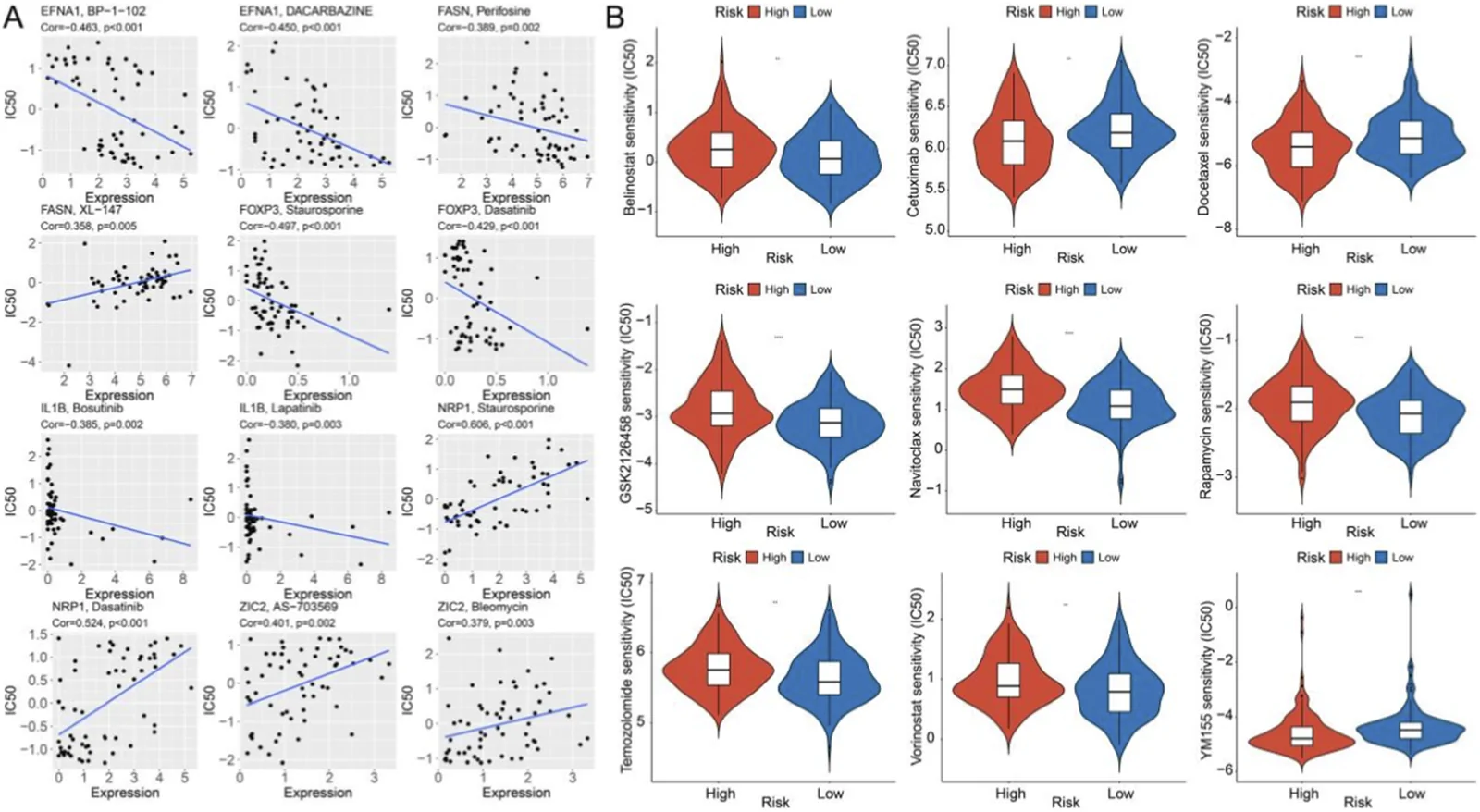
Drug sensitivity prediction based on the lymphangiogenesis-related risk signature. **(A)** CellMiner-based correlation analysis between the expression levels of the six signature genes and anticancer drug sensitivity. **(B)** Predicted drug sensitivity in TCGA-CESC patients estimated by the pRRophetic algorithm in the high- and low-risk groups.

To explore potential therapeutic implications of the six-gene signature, we first queried the CellMiner database to evaluate associations between signature gene expression and anticancer drug sensitivity across the NCI-60 panel. Representative top correlations are shown in Figure 7A. EFNA1 expression was negatively correlated with sensitivity indices for BP-1–102 and dacarbazine (higher EFNA1 associated with higher predicted resistance; Cor <0, P < 0.01). FASN showed drug-dependent associations: FASN expression was negatively correlated with sensitivity to perifosine (Cor <0, P < 0.01) but positively correlated with XL-147 response (Cor >0, P < 0.01). FOXP3 expression was inversely correlated with sensitivity to staurosporine and dasatinib (Cor <0, P < 0.01). IL1B expression was negatively correlated with bosutinib and lapatinib sensitivity (Cor <0, P < 0.01). NRP1 exhibited a positive correlation with dasatinib and staurosporine response (Cor >0, P < 0.01). ZIC2 showed positive correlations with AS-703569 and bleomycin sensitivity (Cor >0, P < 0.01). Collectively, these results suggest that the prognostic genes are linked to heterogeneous drug-response patterns, supporting their potential relevance to therapeutic stratification. Next, using pRRophetic, we estimated the IC50 values of multiple anticancer agents for each CESC patient and compared predicted sensitivity between high- and low-risk groups (Figure 7B). Regarding standard-of-care treatments, no significant differences were observed in the predicted sensitivity to Cisplatin and Paclitaxel between the risk strata (P > 0.05). This suggests that the prognostic value of the signature is independent of conventional chemotherapy response and instead reflects the intrinsic biological aggressiveness and metastatic potential of the tumor. Nevertheless, the high-risk group displayed lower predicted IC50 values for several other agents, including cetuximab, docetaxel, and YM155. Conversely, the low-risk group showed lower predicted IC50 values (higher sensitivity) for belinostat, GSK2126458, navitoclax, rapamycin, temozolomide, and vorinostat.

### 3.8 Construction of a ceRNA regulatory network for the six-gene lymphangiogenesis-related prognostic signature

**FIGURE 8:**
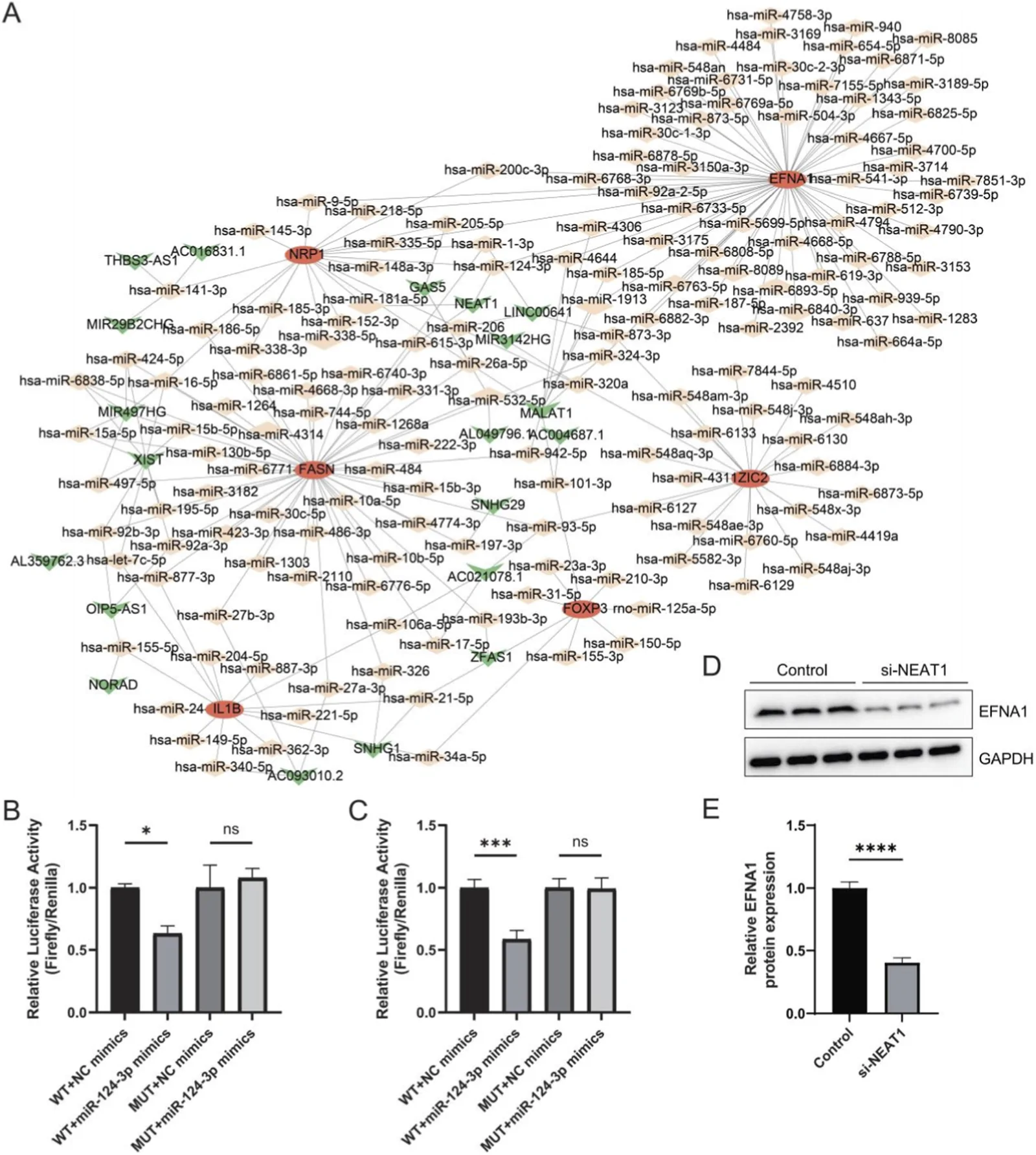
Construction and molecular validation of the ceRNA regulatory network. **(A)** Integrated lncRNA-miRNA-mRNA network. **(B,C)** Dual-luciferase reporter assays in SiHa cells confirming the direct binding of miR-124–3p to EFNA1-3′UTR and NEAT1 .**(D)** Western blot (WB) analysis of EFNA1 protein expression in SiHa cells following NEAT1 knockdown (si-NEAT1). **(E)** Quantitative analysis of WB protein bands showing significant reduction of EFNA1 protein level.

To further explore potential upstream post-transcriptional regulatory mechanisms underlying the six-gene lymphangiogenesis-related prognostic signature, we constructed an lncRNA–miRNA–mRNA ceRNA network (Figure 8A). Network visualization indicated that the six signature genes were connected to multiple miRNAs, suggesting that this prognostic program may be extensively shaped by miRNA-mediated repression. Several signature genes, particularly EFNA1, FASN, and ZIC2, displayed dense miRNA connectivity and occupied hub-like positions, implying increased regulatory complexity and potential vulnerability to upstream non-coding modulation. On the lncRNA layer, widely implicated cancer-associated lncRNAs (e.g., NEAT1, MALAT1, XIST, and NORAD) showed broad interactions with multiple miRNAs and converged on the signature mRNAs, supporting a putative “sponge” mechanism that could indirectly influence lymphangiogenesis-related gene expression in CESC. The construction of this ceRNA network further highlights the potential regulatory value of these non-coding RNA axes, particularly for key hub lncRNAs such as NEAT1 and MALAT1, which have been previously implicated in cervical cancer epithelial-mesenchymal transition (EMT) and metastasis (Xu et al., 2020; Sun et al., 2016).

To transition from bioinformatic prediction to experimental confirmation, we selected the NEAT1/miR-124–3p/EFNA1 axis for molecular validation in SiHa cells. Dual-luciferase reporter assays revealed that miR-124–3p mimics significantly suppressed the luciferase activity of the WT-EFNA1 and WT-NEAT1 groups, while no inhibitory effect was observed in the corresponding MUT groups (Figures 8B,C), confirming the direct binding interactions. Furthermore, Western blot analysis showed that the protein expression of EFNA1 was markedly reduced following the knockdown of NEAT1 (Figure 8D), which was further substantiated by protein quantification (Figure 8E). These findings provide concrete experimental evidence that NEAT1 regulates EFNA1 expression by sponging miR-124–3p, thereby supporting the functional relevance and biological plausibility of our proposed ceRNA framework. Collectively, the ceRNA network provides a systems-level view of non-coding RNA regulation surrounding the prognostic signature and offers a verified regulatory mechanism for future therapeutic exploration.

### 3.9 RT-qPCR validation of key genes in cervical cancer cell lines

**FIGURE 9:**
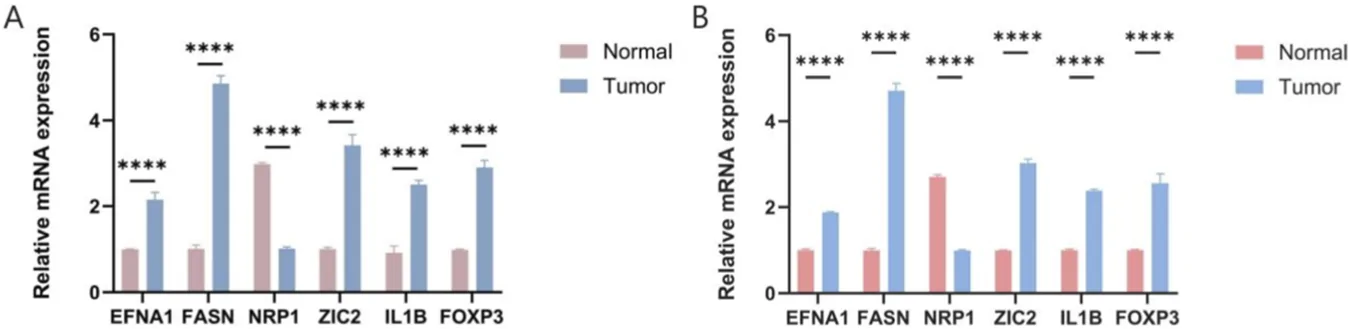
RT-qPCR validation of key genes in cervical cancer and normal cervical epithelial cell lines. The mRNA expression levels of EFNA1, FASN, NRP1, ZIC2, IL1B, and FOXP3 were quantified by RT-qPCR in the human cervical cancer cell line HeLa **(A)** and SiHa **(B)** (Tumor) and the human cervical epithelial cell line H8 (Normal).

To experimentally validate the bioinformatically identified hub genes, we quantified the mRNA expression levels of EFNA1, FASN, NRP1, ZIC2, IL1B, and FOXP3 in both HeLa (adenocarcinoma) and SiHa (squamous cell carcinoma) cell lines compared with the normal cervical epithelial cell line H8. As shown in Figure 9A, the expression patterns in HeLa cells were consistent with our predictions. To satisfy the need for generalizability across histologic subtypes, we repeated the validation in SiHa cells. As shown in Figure 9B, EFNA1, FASN, ZIC2, IL1B, and FOXP3 were significantly upregulated in SiHa cells, while NRP1 was significantly downregulated compared with H8 cells (P < 0.0001). Collectively, these results provide robust experimental support across both major histologic subtypes of cervical cancer.

## 4 Discussion

In this study, we developed a six-gene signature centered on lymphangiogenesis and demonstrated its strong prognostic value in cervical squamous cell carcinoma and CESC. The signature stratified patients into high-risk and low-risk groups with significantly different overall survival. On multivariate analysis, the risk score remained an independent predictor of survival even after adjusting for traditional factors such as stage, highlighting that the signature captures unique prognostic information (Deng et al., 2024). Notably, the concordance index (C-index) and time-dependent ROC analyses in our cohort showed that this six-gene model outperformed individual clinicopathological variables in predicting outcome, echoing findings from prior gene-signature studies (Li S. et al., 2025). This underscores a key point: molecular features of the tumor—in this case, expression of lymphangiogenesis-related genes—can refine risk stratification beyond anatomical staging. Our results align with Liu et al. (2022), who reported that a hypoxia/angiogenesis gene score was a superior survival predictor compared to TNM stage in cervical cancer (Liu et al., 2022). Likewise, recent work by Guo et al. (2023) confirms that even a small gene panel related to nodal metastasis can significantly distinguish high vs. low risk patients (Guo et al., 2023). By specifically focusing on lymphatic vessel formation pathways, our six-gene signature addresses a niche that previous models did not: it serves as a proxy for the tumor’s lymphangiogenic and metastatic potential, which is directly tied to prognosis in CESC.

It is worth noting that our signature’s strength lies not just in statistical performance but in biological coherence. All six genes in the model have plausible links to cancer progression, and several have documented roles in lymphangiogenesis or metastasis. For example, our signature includes FASN (fatty acid synthase), a metabolic oncogene recently shown to drive cervical cancer invasion and lymphatic spread via secretion of pro-lymphangiogenic factors (PDGF-AA and IGFBP-3) (Martínez-Ramírez et al., 2026; Zhou et al., 2025). High FASN expression correlates with increased micro-lymphatic vessel density in tumors and independently predicts poor survival (Du et al., 2022). Another signature gene, PRDX3, has been implicated in promoting lymph node metastasis in cervical cancer by enhancing anoikis resistance and activating NF-κB signaling; overexpression of PRDX3 in primary tumors strongly associates with nodal positivity and worse outcomes (Wen et al., 2025). The biological rationality of the signature is further supported by the specific roles of FOXP3 and IL1B in the lymphatic microenvironment. IL1B acts as a potent pro-inflammatory cytokine that promotes a lymphangiogenic microenvironment partly through induction of VEGF-C signaling (Morfoisse et al., 2014). Meanwhile, FOXP3+ regulatory T cells contribute to an immunosuppressive niche through TGF-β-mediated signaling, which has been implicated in lymphatic vessel remodeling, lymphangiogenesis, and tumor lymphatic dissemination (Itoh and Watabe, 2022). These concordant findings from distinct studies reinforce the relevance of our chosen genes to the clinical behavior of CESC. In short, the signature is prognostic because it encapsulates key drivers of tumor aggressiveness.

Beyond risk stratification, our analysis reveals that the six-gene signature is linked with distinctive tumor immune microenvironments and subtype characteristics. Tumors classified as high-risk by our model tended to exhibit an immunologically “cold” or immunosuppressive profile. Specifically, we observed lower infiltration of CD8^+^ T cells and NK cells and higher presence of M2-polarized macrophages and regulatory T cells in high-risk tumors. This mirrors patterns seen with other pro-metastatic gene signatures: Kang et al. (2022) found that cervical cancers with high angiogenesis scores had reduced expression of multiple immune checkpoint genes (PD-1, CTLA-4, *etc.*) and overall lower tumor immunogenicity (Kang et al., 2022), suggesting a more immuno-evasive phenotype. In our study, the high lymphangiogenesis-risk group similarly showed evidence of T-cell dysfunction, including upregulation of checkpoint molecules like PD-L1 and TIM-3. Paradoxically, some metrics of immune infiltration were elevated in high-risk tumors (e.g., a higher proportion of exhausted CD8^+^ T cells), indicating that these tumors are not completely devoid of immune cells but likely actively suppress effective anti-tumor immunity. This observation is consistent with Guo et al. (2023), who reported that their high-risk (LNM-linked) group had increased immune checkpoint expression despite worse survival, implying those tumors induce an ineffective immune response that could potentially be leveraged by immunotherapy (Guo et al., 2023). We postulate that an active lymphangiogenic program in the tumor might contribute to immune escape. Lymphatic endothelial cells can scavenge antigens and modulate dendritic cell trafficking, dampening anti-tumor immune activation (Tamburini et al., 2014; He et al., 2021). Moreover, pro-lymphangiogenic factors like VEGF-C are known to directly create an immunosuppressive milieu by recruiting macrophages and inducing T cell tolerance in tumor contexts (Lund et al., 2012; Tacconi et al., 2019; Cousin et al., 2021). Our finding that VEGFC (encoding VEGF-C) is elevated in the high-risk group (itself not in the final six-gene panel due to Cox selection, but enriched in high-risk tumors) supports this mechanistic link. Furthermore, our findings suggest a critical crosstalk between immune patterns and lymphangiogenesis. In high-risk tumors, the high expression of lymphangiogenesis-related genes may facilitate immune evasion by exporting tumor-infiltrating lymphocytes or attracting myeloid-derived suppressor cells (MDSCs) through expanded lymphatic channels, ultimately leading to the observed ‘cold tumor' phenotype and poor prognosis.

Our findings resonate with a growing body of literature emphasizing the prognostic and therapeutic importance of tumor vasculature and microenvironment in cervical cancer. Angiogenesis (formation of blood vessels) has long been a target in CESC, with the anti-VEGF-A antibody bevacizumab improving survival in advanced cases (Tewari et al., 2014; Manso et al., 2024). However, lymphangiogenesis has received comparatively less attention, despite evidence that it is crucial for metastasis and is associated with poor outcomes (Pepper, 2001; Zhao et al., 2021). Karakousi et al. (2024) noted in a comprehensive review that tumors often co-opt lymphatic vessels to facilitate spread and immune evasion, and that high lymphatic vessel density frequently predicts metastasis in multiple cancers (Karakousi et al., 2024). Our six-gene signature provides concrete genomic evidence supporting this concept in CESC. It also complements recent gene signatures derived for related purposes. For instance, a 2023 study by Zhou et al. (Front. Immunol.) classified cervical cancers by m^6A^ RNA methylation patterns and noted that m^6A^-regulator-defined subtypes differed in immune infiltration and survival (Mao et al., 2023). Intriguingly, ALKBH5, an m^6A^ demethylase, emerged in our lymphangiogenesis signature, suggesting a convergence between epitranscriptomic regulation and lymphatic metastasis. ALKBH5 has been highlighted as a context-dependent prognostic factor in several malignancies and was very recently shown to be upregulated by HPV oncoprotein E7 in cervical cancer (Li C. et al., 2025). Functionally, ALKBH5 promotes cervical tumor progression by demethylating m^6A^ on transcripts like PAK5, thereby stabilizing them and enhancing pathways that drive cell invasion and metastasis (Huo et al., 2023). Liang et al. (2022) likewise demonstrated that ALKBH5-mediated m^6A^ modification of a circular RNA (circCCDC134) led to HIF-1α upregulation and increased cervical cancer metastasis (Liang et al., 2022). These studies corroborate our result that ALKBH5 (as part of the signature) is associated with aggressive, metastasis-prone disease. They also hint at why ALKBH5 might correlate with lymphangiogenesis: by stabilizing pro-metastatic mRNAs (HIF1A, PAK5, etc.), ALKBH5 can induce a hypoxia-like program and EMT, which often includes VEGF-C upregulation and lymphatic invasion (Dong et al., 2021; Xu et al., 2024; Ji et al., 2023). Furthermore, emerging evidence links ALKBH5 to the immune microenvironment—it can modulate cytokine expression and checkpoint molecule levels, thus affecting T cell infiltration and function (Li C. et al., 2025). In summary, the inclusion of ALKBH5 in our model bridges novel epigenetic mechanisms with classical metastatic pathways. This cross-talk exemplifies how our signature not only has prognostic utility but also provides mechanistic clues consistent with recent cervical cancer research.

While our six-gene signature shows significant prognostic potential, several limitations warrant consideration. First, the retrospective nature of the TCGA and the relatively small size of the GSE52903 cohort (n = 51) necessitate further validation in large-scale, prospective multi-center studies to refine clinical risk cutoffs; however, the successful cross-platform validation (microarray vs. RNA-seq) underscores the model’s robust generalizability. Second, the current model does not incorporate treatment-specific outcomes, such as individual responses to chemoradiation or immunotherapy. Finally, transitioning from transcriptomic profiling to practical clinical assays, such as simplified IHC or RT-qPCR panels for key signature members like FASN or VEGFA, remains a crucial step for future diagnostic implementation in routine pathology laboratories.

In summary, the discussion above illustrates that our lymphangiogenesis-related six-gene signature is not only a robust prognostic tool but also a window into the biology of cervical cancer metastasis and immunity. By contextualizing our findings within existing literature, we see a convergent narrative: tumors that are primed to spread via lymphatics share features of hypoxic signaling, immune escape, and certain metabolic or epigenetic alterations. Our work reinforces this narrative and pushes it a step further by providing a tangible gene set that embodies these features. This lays the groundwork for both improved patient stratification and novel therapeutic interventions targeting the lymphangiogenic and immunologic underpinnings of cervical cancer.

## 5 Conclusion

In conclusion, we developed a lymphangiogenesis-related six-gene signature that reliably stratifies CESC patients by survival risk beyond conventional clinicopathological factors. High-risk tumors were associated with more aggressive biology, including enhanced lymphangiogenic activity, EMT/hypoxia-related programs, and an immunosuppressive microenvironment, whereas low-risk tumors showed better outcomes. The signature also suggests potential therapeutic opportunities, including targeting lymphangiogenesis- and metabolism-related pathways and prioritizing immunomodulatory strategies in high-risk disease; ALKBH5 further implicates a possible role of m^6A^ regulation. Prospective validation and development of a practical assay are needed to enable clinical translation.

## Data Availability

The original contributions presented in the study are publicly available. This data can be found in the Gene Expression Omnibus (GEO) repository with the accession number GSE52903.
