# Supplementary material for: Lymphangiogenesis-related gene signature–based risk model for prognostic assessment of cervical cancer: immune–metabolic characterization and molecular subtype analysis
Source: Front Genet. 2026 Aug 31;17:1835653. doi: 10.3389/fgene.2026.1835653 (PMC13571821; doi:10.3389/fgene.2026.1835653)
Supplement: Supplementary file 1 [file DataSheet1.docx]

**Supplementary data**

**Supplementary figure 1. Correlation analysis of ALKBH5 expression with signature genes.**

**Supplementary figure 2. Association of the RiskScore with lymph node metastasis (LNM) and lymphovascular space invasion (LVSI).**
